# Pulsed-wave Ultrasound Hyperthermia Enhanced Nanodrug Delivery Combined with Chloroquine Exerts Effective Antitumor Response and Postpones Recurrence

**DOI:** 10.1038/s41598-019-47345-8

**Published:** 2019-08-28

**Authors:** Chi-Feng Chiang, Yu-Hone Hsu, Chih-Chun Liu, Po-Chin Liang, Shi-Chuen Miaw, Win-Li Lin

**Affiliations:** 1 Department of Biomedical Engineering, National Taiwan University, Taipei, Taiwan; 2 Division of Neurosurgery, Department of Surgery, Kaohsiung Veterans General Hospital, Kaohsiung, Taiwan; 3 Graduate Institute of Immunology, College of Medicine, National Taiwan University, Taipei, Taiwan; 4 Department of Radiology, Department of Medical Imaging, National Taiwan University Hospital, Taipei, Taiwan; 5 Institute of Biomedical Engineering and Nanomedicine, National Health Research Institutes, Miaoli, Taiwan

**Keywords:** Cancer microenvironment, Chemotherapy, Cancer metabolism

## Abstract

Autophagy is found to serve as a surviving mechanism for cancer cells. Inhibiting autophagy has been considered as an adjuvant anti-cancer strategy. In this study, we investigated the anti-tumor effect of combining pulsed-wave ultrasound hyperthermia (pUH) enhanced PEGylated liposomal doxorubicin (PLD) delivery with an autophagy inhibitor chloroquine (CQ). BALB/c mice bearing subcutaneous 4T1 tumor received intravenous injection of PLD (10 mg/kg) plus 15-minute on-tumor pUH on Day 5 after tumor implantation and were then fed with CQ (50 mg/kg daily) thereafter. Prolonged suppression of tumor growth was attained with PLD + pUH + CQ treatment, whereas in PLD + pUH group tumors quickly recurred after an initial inhibition. Treatment with CQ monotherapy had no benefit compared to the control group. Immunohistochemical staining and Western blotting showed that autophagy of cancer cells was blocked for the mice receiving CQ. It indicates that PLD + pUH + CQ is a promising strategy to treat cancer for a long-term inhibition.

## Introduction

Autophagy is a catabolic process that turns over old proteins and organelles, allows cells to recycle cellular components and provides required energy^1,2^. It is a key component in maintaining homeostasis of cellular environment. Autophagy also plays a crucial role in cancer pathophysiology. It is believed that autophagy prevents cancer development, but helps cancer cells within an already established tumor to survive from stress and threats^1–6^. Therefore, autophagy inhibition has been investigated as an evolving strategy to fight against advanced cancer^5–7^.

Chloroquine (CQ) and its analogue hydroxychloroquine (HCQ) are widely used anti-malarial drugs. They inhibit lysosomal acidification and hence block the formation of autophagosome as well^5^. In addition to autophagy inhibition, CQ was reported to possess multiple mechanisms to fight against cancer, including targeting against cancer stem cells (CSC)^8^ and inducing tumor vessel normalization^9^. The link between CQ and cancer can be traced to 1980s, a malaria suppression program carried out in Tanzania utilizing CQ were found accompanied with a significant decline in Burkitt’s lymphoma within the period of CQ distribution program^10^. There were several ongoing clinical trials using CQ (or HCQ) additional to conventional chemotherapeutic agents to treat various kinds of cancer, including colorectal cancer, glioblastoma, and pancreatic cancer^6^.

Hyperthermia has been used to treat many kinds of cancer for decades, either monotherapy or in combination with other anti-cancer therapy. The synergistic effect between hyperthermia and chemotherapeutic agents such as doxorubicin was well-known^11^. Furthermore, localized hyperthermia increases blood flow and vascular permeability in the heated tumor region, and therefore enhances the delivery of nanodrug into cancer cells^12^. Ultrasound was investigated as a modality to induce hyperthermia and was found advantageous over other thermal sources for its noninvasiveness and penetrating ability into deep tissues^13^.

Previous studies^14,15^ showed that short-time pulsed-wave ultrasound hyperthermia (pUH) not only enhanced the delivery of PEGylated liposomal doxorubicin (PLD) but also elicited inhibitory effect directly on cancer cells. However, in some cases the cancer tumors were macroscopically destroyed by the combined therapy of PLD + pUH, they still recurred several days or weeks after the treatment.

**Figure 1:**
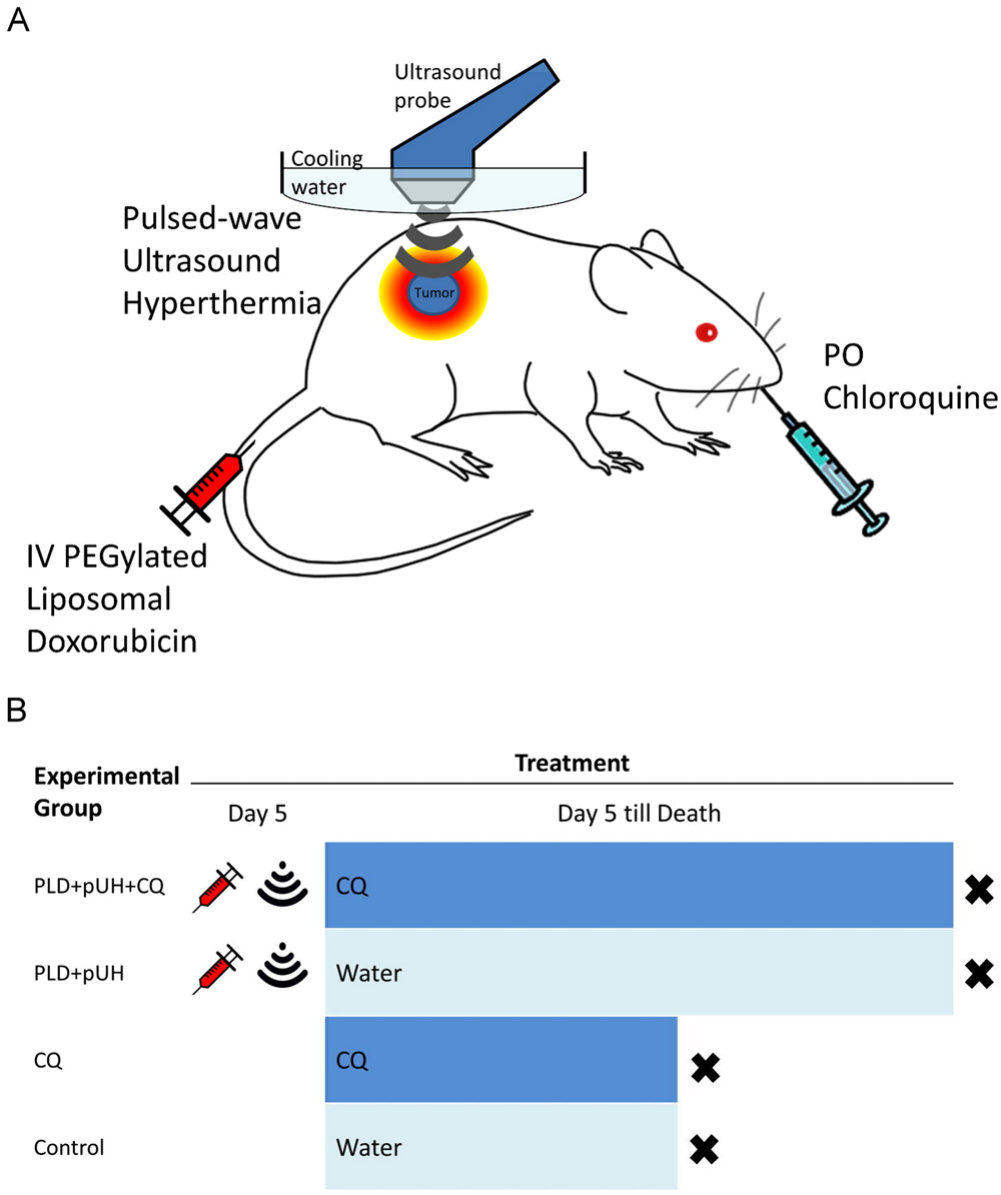
(**A**) The scheme of PEGylated Liposomal Doxorubicin (PLD) + pulsed-wave Ultrasound Hyperthermia (pUH) + chloroquine (CQ) in cancer treatment. (**B**) Time schedule of treatment experiement. PEGylated Liposomal Doxorubicin (PLD) was given intravenously on Day5 after tumor implantation. Pulsed-wave ultrasound hyperthermia (pUH) was administered 10~15 minutes after PLD administration. Then mice were orally fed chloroquine (CQ) dissolved in drink water daily till experiment end.

The aim of present study was to investigate an effective strategy to successfully treat tumor, prevent tumor recurrence, and improve the disease-free survival. We evaluated the combination treatment of PLD + pUH with long-term CQ administration to see if it successfully suppressed tumor growth and prevented recurrence. The concept of therapy and design of experiment was schemed in Fig. 1A,B, respectively. We found that pUH enhanced PLD delivery in combination with CQ could long-term suppress 4T1 tumor growth and postpone its recurrence.

## Materials and Methods

### Chemical reagents

The PEGylated liposomal doxorubicin (PLD) used in this study was a commercial product, Lipo-Dox® purchased from Taiwan Tung Yang Biopharm Company Ltd. It contains 2 mg/mL doxorubicin and 14 mol/mL phospholipids. The lipid composition is distearoylphosphatidylcholine, cholesterol, and PEG-DSPE with a molar ratio 3:2:0.3, respectively. The average particle diameter of the PLD determined by dynamic light scattering is 84.5 ± 18.6 nm at 37 °C, with a narrow size distribution. The mean particle diameter, size distribution, and doxorubicin release rate were quite unaffected by temperature^16^. The elimination half-life of Lipo-Dox is about 65 h. Chloroquine (CQ) was purchased from Sigma-Aldrich.

### Tumor cells

4T1 murine breast cancer cells (ATCC® CRL-2539TM) were cultured in Roswell Park Memorial Institute (RPMI) 1640 medium supplemented with 10% heat-inactivated fetal bovine serum (FBS) in 10 cm tissue culture dishes in a 5% CO_2_-containing incubator at 37 °C.

### MTT cytotoxicity assay

MTT cytotoxicity assay was used to analyze the cytotoxic effect of different treatment on cancer cells. 4T1 murine breast cancer cells were seeded on a 96-well plate at a density of 2.5 × 10^3^ cells/well, supplied with 100 µL RPMI 1640 medium containing 10% fetal bovine serum. After 24 hours incubation, the cells were subjected to assigned treatment conditions. The concentration of PLD ranged from 0.02 mg/mL to 1 mg/mL. The concentration of CQ was 10 µM. Hyperthermia was carried out with 43°C water bath for 5 mimutes. Cells incubated without any treatment were used as the blank control group. After 48 hours of treatment, old drug-containing medium was removed, and 3-(4,5-dimethylthiazol-2-yl)-2,5-diphenyl tetrazolium bromide solution (5 mg/mL) was added (40 µL per well) and incubated for further 90 minutes. Then 3-(4,5-dimethylthiazol-2-yl)-2,5-diphenyl tetrazolium bromide solution was removed, and precipitated crystals were dissolved with dimethylsulfoxide (DMSO, 150 µL per well). The absorbance values were determined with a microplate reader (Synergy HTX; BioTek Instruments Inc., Winooski, VT, USA) at a wavelength of 540 nm.

### *In vivo* tumor model

All animal experiments were approved by the Institution of Animal Care and Use Committee of College of Medicine in National Taiwan University. All procedures regarding animal experiment were conducted in accordance to relevant guidelines and regulations. Eight-week-old female BALB/c mice were acquired from BioLASCO Taiwan Co., Ltd. and were housed with a 12-h light/dark cycle and allowed free access to water and standard diet. The mice were anesthetized by 1–3% isoflurane inhalation during the tumor implantation procedure. The hair on right flank was shaved before tumor implantation. A total of 10^6^ 4T1 tumor cells suspended in 100 μL of phosphate buffered saline (PBS) were slowly injected subcutaneously into the right flank of the mice.

### Animal treatment experiment

The time schedule of treatment experiment was summarized in Fig. 1B. Five days after tumor implantation, the tumor volume reached about 100 mm^3^ and the treatment started. The tumor-bearing mice were randomly assigned to one of the following four groups: PLD + pUH + CQ, PLD + pUH, CQ, and Control. PLD solution was diluted with normal saline (1:1 volume ratio) and then injected via tail vein with a dose of 10 mg/kg body weight according to the body weight of individual mouse measured right before the treatment. Ten minutes later, pUH was applied with an ultrasound sonicator (US-700; ITO, Japan) at 1 MHz frequency, 50% duty cycle, 3 W/cm^2^ intensity, and 15 minutes sonication duration. The ultrasound probe was immersed in a home-made water bag filled with degassed water, and the water bag was mounted onto the tumor to prevent skin burn during ultrasound hyperthermia. CQ (dose 50 mg/kg body weight, dissolved in drinking water) was given daily via oral gavage since Day 5. Mice were closely monitored for their health status, and tumors were measured with a digital caliper every three days. Tumor volume was estimated by 0.5 * Length * Width^2^.

### Histopathological examination and immunohistochemical study

The mice were subcutaneously implanted with 10^7^ 4T1 murine breast cancer cells suspended in 100 μL of PBS at right flank. Seven days later, the mice were divided into the following four groups: PLD + pUH + CQ, PLD + pUH, CQ, and Control, and then received treatment. The treatment procedures were similar to description in the Animal treatment experiment section, with following change: CQ was given daily via oral gavage with a dose of 100 mg/kg body weight. Three days after the treatment, the mice were euthanized and the tumors were harvested. The harvested tumors were cut into two halves. One was subjected to histopathological examinations, immunohistochemical studies, and TUNEL assay. The other was processed for Western blotting.

Halves of the harvested tumors were fixed in 4% paraformaldehyde solution for three days and then embedded in paraffin blocks and sliced. Tumor tissue sections were stained with hematoxylin-eosin (H&E) for histopathological examination. For immunohistochemical studies, tumor tissue sections were de-paraffined and rehydrated, and then treated with Proteinase K in a 37 °C incubator to retrieve antigens. After antigen retrieval, tissue sections were incubated with primary antibody against LC3B (1:500, CellSignaling, cat. No. 3868) overnight at 4 °C. After several washes, tissue sections were incubated with Dako LSAB 2 system to label primary antibodies and counterstained with hematoxylin for greater contrast. After dehydration and mounting, the prepared tissue sections were examined with microscope.

### TUNEL assay

Tumor tissue sections were processed for TUNEL assay using a DeadEnd Fluorometric TUNEL system (Promega, Madison, WI, USA) according to the manufacturer’s instructions. In brief, tissue slides were fixed with 4% paraformaldehyde solution and permeabilized with 20 μg/mL proteinase K. The slides were then labeled with rTdT enzyme mixture overnight at 37 °C. The slides were then stained with Hoechst 33342 dye and mounted. Fluorescence images were obtained using a confocal microscope (AxioImager M1; Carl Zeiss Ltd., Oberkochen, Germany), using the FITC channel for detecting apoptotic cells and DAPI channel (excitation at 340–380 nm and emission at 435–485 nm) for revealing cell nuclei, respectively. All images were captured using the same exposure time. The pictures were merged using AxioVision Rel. 4.8 software (Carl Zeiss Ltd., Oberkochen, Germany).

To quantitatively compare the difference of apoptosis activity among groups, five representative fields were picked from each slide, and the total fluorescent intensity in each field was measured with ImageJ software. Statistical significance was tested with Student t test.

### Western blotting

The tumor tissues were homogenized and purified with RIPA and then prepared with 6X SDS sample buffer. Equal aliquots of protein (30 μg) were loaded onto 10% SDS-PAGE electrophoresis gels and run with 100 V for 60 min. The proteins were then transferred to nitrocellulose membranes with 100 V for 100 min. The membranes were then washed with TBST for several times and blocked for 60 min. After several washes, the membranes were cut into corresponding pieces and incubated with anti-LC3B antibody (1:1000, CellSignaling, cat. No. 3868), anti-β-actin antibody at 4 °C overnight. After incubation, anti-LC3B antibodies were labeled by incubating with goat anti-rabbit HRP antibody (Santa Cruz) for 60 min at room temperature, whereas anti-β-actin antibodies were labeled by incubating with HRP goat anti-mouse antibody (BioLegend Cat. No. 405306). The expression of proteins was visualized with WesternBright^TM^ ECL HRP substrate (Advansta Inc., CA, USA). The quantification of bands was performed with ImageJ software.

### Statistical analysis

All numerical values were expressed in the form of mean ± standard error of mean (SEM). The results were analyzed with two-way Student t test, and statistical significance was defined as p < 0.05. Analysis was performed with IBM SPSS Statistics version 20.0 (IBM Corp., Armonk, NY, USA).

## Results

### MTT cytotoxicity assay

**Figure 2:**
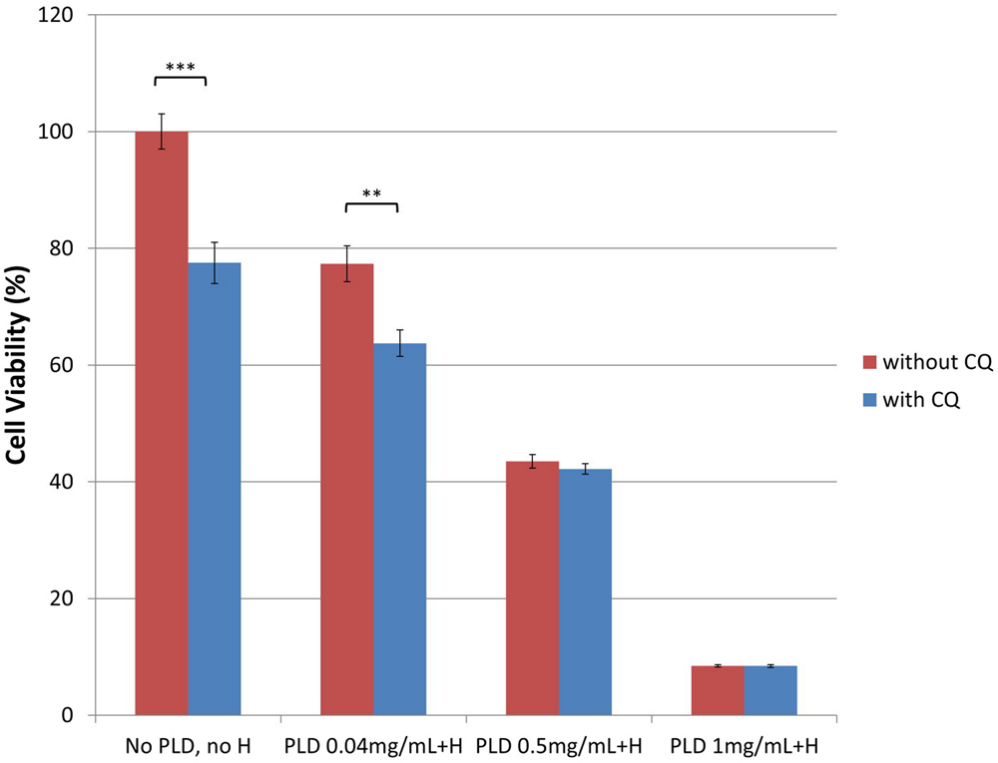
MTT Cytotoxicity Assay. The cell viability was reduced by PLD with hyperthermia in a dose-dependent manner. The addition of CQ (10 μM) further potentiated the cytotoxicity of PLD + H comparing to the counterpart without CQ. **p < 0.01, ***p < 0.001. Abbreviation: H = hyperthermia.

The cytotoxicity induced by different treatments was determined with MTT assay, and the result was represented in Fig. 2. It was shown that treatment with PLD plus hyperthermia reduced cell viability in a dose-dependent fashion. By adding CQ, the cell viability was further reduced in all treatment conditions as compared to their corresponding counterparts without CQ. The differences were more remarkable in low-dose PLD groups, but not in high-dose PLD groups.

### Combination treatment of PLD + pUH and CQ inhibited cancer tumor growth and delayed its recurrence

**Figure 3:**
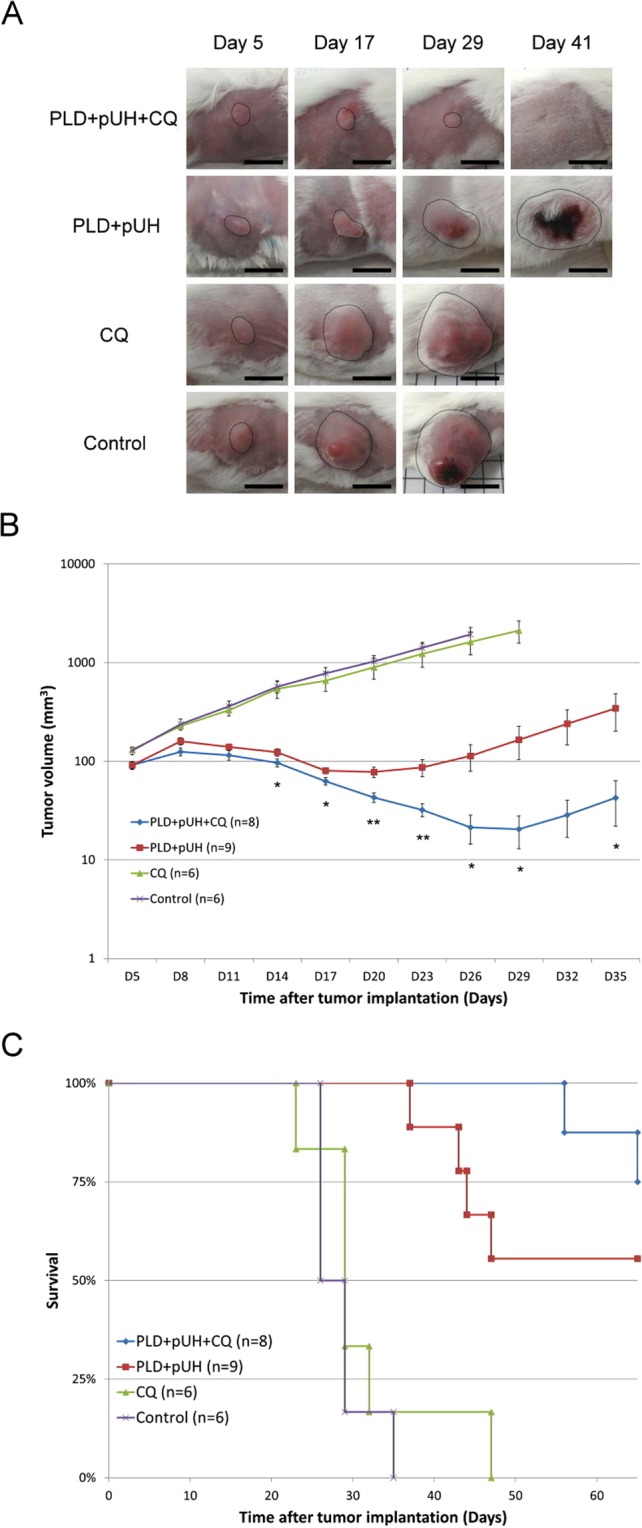
Tumor growth and survival. (**A**) Representative photographs of tumor for each group. Region encircled by dashed line indicated tumor. Scale bar = 1 cm. (**B**) The response of subcutaneous 4T1 murine breast cancer to different treatment: PLD + pUH + CQ, PLD + pUH, CQ, and control groups. *Denotes p < 0.05, and **denotes p < 0.01 between PLD + pUH + CQ and PLD + pUH, respectively. (**C**) The Kaplan-Meier survival plot for PLD + pUH + CQ, PLD + pUH, CQ, and control groups.

To investigate the effect of combination treatment of PLD + pUH with CQ, a single treatment of PLD + pUH was applied on 4T1 tumor-bearing mice on Day 5 followed by daily-given CQ. Figure 3B showed that tumor growth in PLD + pUH group was inhibited, and the tumor shrank after the treatment since Day 5. However, the tumors treated with PLD + pUH were then relapsing since Day 17. On the contrary, treatment with PLD + pUH plus daily CQ administration not only inhibited tumor growth, but also drastically delayed tumor relapse until Day 32. Additionally, the growth rate of recurring tumors in the PLD + pUH + CQ group was much slower than that in the PLD + pUH group. Monotherapy with CQ alone had no benefit in inhibiting tumor growth as compared to the control group. All therapeutics were well tolerated, as neither significant drop in body weight nor apparent change in general activity was observed.

As shown in Fig. 3C, the survival rate of the PLD + pUH + CQ group seemed better than the PLD + pUH group. Up to Day 60, there were 6 among 8 treated mice achieved complete remission (defined by no observable tumor) in the PLD + pUH + CQ group, and 5 among 9 treated with PLD + pUH achieved complete remission. Nonetheless, the survival advantage of PLD + pUH + CQ group over PLD + pUH group did not reach statistical significance (p-value: 0.2). The survival of the CQ group did not differ from the control group, showing that CQ monotherapy had no significant benefit in suppressing tumor growth.

### Immunohistochemical study proved autophagy of tumor cells blockaded by CQ administration

**Figure 4:**
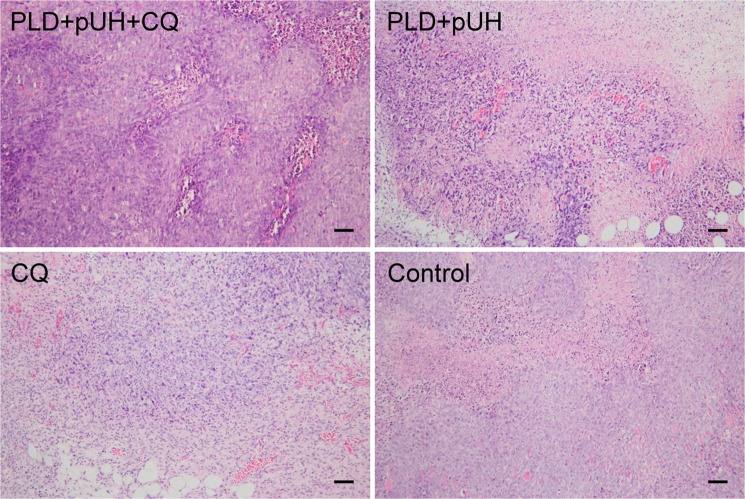
Histological examinations with hematoxylin-eosin staining for each experimental group. Scale bar = 100 μm.

**Figure 5:**
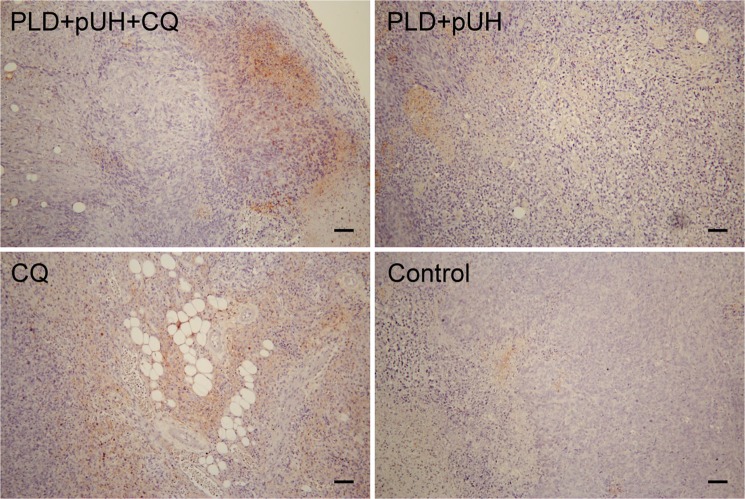
Immunohistochemical stain for LC3 (brown stain) for each experimental group. LC3 accumulation reflects late-stage inhibition of autophagy. Greatly increased accumulation of LC3 was observed in both PLD + pUH + CQ and CQ groups, whereas slightly increase in the PLD + pUH group, and nearly no accumulation in the control group. Scale bar = 100 μm.

To investigate the change in tumor tissue affected by PLD + pUH + CQ treatment, tumor-bearing mice received single treatment of PLD + pUH on Day 7 after tumor implantation and were then fed daily with high dose CQ solution. Three days later, tumor were harvested and subjected to histopathological studies. H&E staining of tumor samples revealed that there was extensive necrosis in all groups (Fig. 4). Some fat cells surrounding the tumors or even infiltrating into tumor stroma could be observed. Figure 5, immunohistochemical staining, shows that LC3 expression (brown color) was markedly enhanced in both PLD + pUH + CQ and CQ groups, slightly increased in the PLD + pUH group, and nearly no expression in the control group. The results correlated well with the expected effect of CQ which inhibited autophagy and resulted in the accumulation of LC3-II.

### TUNEL assay showed apoptosis increased by PLD + pUH, not by CQ

**Figure 6:**
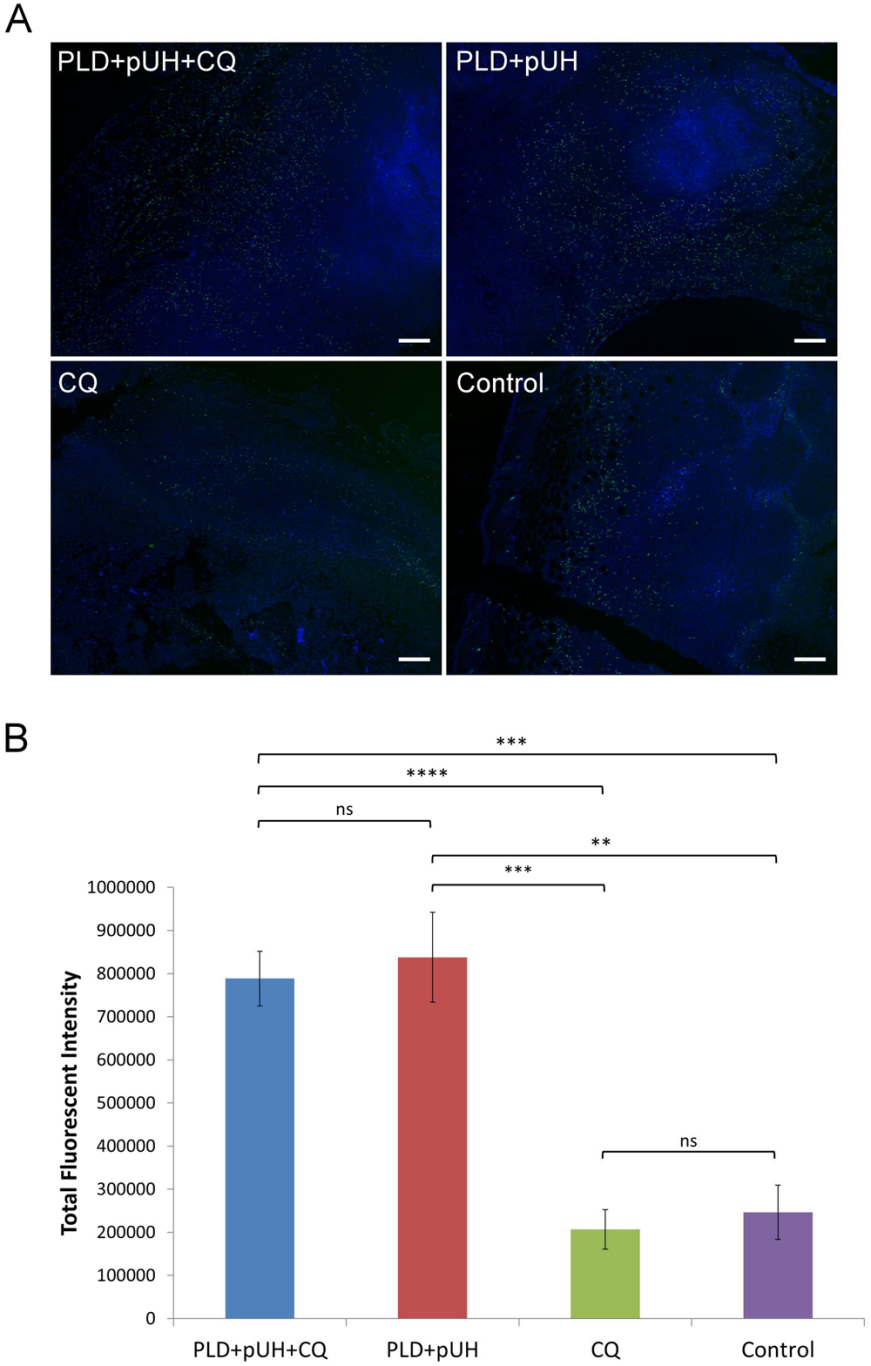
(**A**) Fluorescent microscopic images of TUNEL assay for each experimental group. Apoptotic signals (green) were much more enhanced in PLD + pUH + CQ group and PLD + pUH group. Scale bar = 200 μm. (**B**) The fluorescent intensities for each experimental group were quantified and analyzed for statistical significance. **p < 0.01. ***p < 0.001. ns: not significant.

TUNEL assay was performed to assess the effects of different therapeutics on apoptosis of the treated tumors. Figure 6A showed that there were apparently stronger signals of apoptosis (green dots) in the PLD + pUH + CQ and PLD + pUH groups than those in the CQ and control groups, and the differences were highly significant (Fig. 6B). The intensities of apoptosis signal were statistically similar in the CQ group and the control group. The difference between PLD + pUH + CQ group and PLD + pUH group was not significant as well. These results reflected that cancer cell apoptosis was induced by the treatment of PLD + pUH. Daily CQ administration did not alter the detectable density of cancer cell apoptosis, meaning CQ solely had no direct pro-apoptotic effect.

### Western blotting

**Figure 7:**
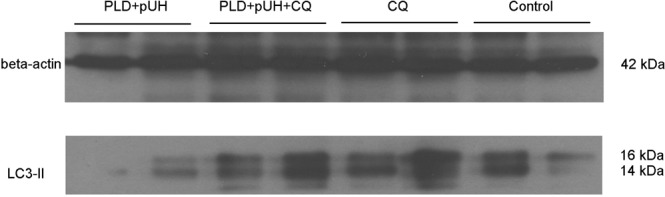
Western blot for LC3 for each experimental group. Increased expression of LC3-II was observed in PLD + pUH + CQ group and CQ group, reflecting the late-stage autohphagy inhibition by CQ. LC3 expression was slightly reduced in PLD + pUH group comparing to control group. Full-length Western blot image is presented in Supplementary Fig. S1.

To demonstrate the autophagy inhibition induced by CQ, Western blotting assay was used to detect LC3 in tumor supernatant. Figure 7 showed the result of Western blotting targeting LC3. The expression of LC3-II was raised in the PLD + pUH + CQ and CQ groups, but not in the PLD + pUH and control groups. These findings coincided with the autophagy inhibition caused by CQ administration observed in immunohistochemical stain.

## Discussion

Previous studies^14,15^ showed that PLD in combination with pUH significantly inhibited the growth of 4T1 murine breast cancer in an *in vivo* brain metastatic tumor model. It was proved that pUH on the tumor after injection of PLD significantly increased the accumulation of PLD in the sonicated tumor tissue and cancer cells. Based on these results, we investigated if inhibition of tumor growth and tumor relapse could be further improved by combining PLD + pUH with CQ. To have a better observation, a subcutaneous tumor model was used instead. The experimental results shown in Fig. 3 displayed that both PLD + pUH with and without CQ could successfully retard 4T1 tumor growth right after the treatment. Stronger retarding effect on tumor growth was obtained with the administration of CQ. The tumor inhibitory effect in the PLD + pUH group did not persist long, as the treated tumor began to grow again since Day 17. Treatment with PLD + pUH destroyed most cancer cells and resulted in tumor shrinkage, but still a small fraction of damaged cancer cells survived and regained the tumor growth several days after the treatment. The story tremendously differed in the case with PLD + pUH treatment followed by daily CQ administration. Treatment with PLD + pUH + CQ not only retarded cancer tumor growth more effectively, but also postponed the tumor regrowth until Day 32 and the regrowth rate of tumor was much slower as compared with the PLD + pUH group. This significant difference suggested that CQ, by blocking the escape mechanism of autophagy, rendered the cancer cells damaged by PLD + pUH treatment more difficult to survive. The prolonged retardation on tumor growth resembled the results obtained by Hirsch *et al*.^17,18^, in which they showed metformin preferentially targeted CSC and exerted long-term inhibitory effect when co-administered with doxorubicin. It implied that the recurrence-postponing effect of CQ observed in our study might be partially attributed to similar anti-CSC activity.

Several researchers claimed that CQ potentiates anti-cancer therapeutics through mechanisms outside autophagy inhibition. Maes *et al*. found that CQ normalized tumor vessel structure, and the effect could not be mimicked by genetic inhibition of autophagy^9^. Balic *et al*. indicated that CQ suppressed CSC via inhibition of CXCR4 and Hedgehog signaling^8^. King *et al*. proposed that CQ synergized mTORi through mechanism related to cholesterol metabolism^19^. Nonetheless, the majority of researches still attributed the anti-tumor potency of CQ to its lysosomal acidifying/autophagy inhibition ability. Choi *et al*. concluded the anti-CSC activity of CQ was through autophagy inhibition despite their finding that Jak2-STAT3 pathway might also play a role^20^. Wei *et al*. showed in their study that autophagy inhibition rendered CSC susceptible to photodynamic therapy, regardless pharmacologic inhibition with CQ or genetic silencing^21^. Likewise, Lee *et al*. also found that CQ sensitized glioma cells to temozolomide, and the sensitizing effect was observed with other autophagy inhibitors as well^22^. They also demonstrated the sensitization by autophagy inhibition was p53 dependent. Similarly, Maycotte *et al*. identified certain subtypes of triple-negative breast cancer more responsive to autophagy-inhibiting treatment, and this susceptibility could be predicted by high STAT3 expression^23^. Furthermore, it is indicated that some activity of CQ shares the same underlying mechanism with autophagy inhibition: both are consequences of lysosome disruption. Elliot *et al*. found that lysosomal inhibition by CQ impaired de novo nucleotide biosynthesis and depleted aspartate in pancreatic ductal adenocarcinoma^24^. Even the aforementioned vessel-normalizing ability of CQ was proposed in a recent study to be related to lysosomal dysfunction^25^.

In immunohistochemical studies and Western blotting, the accumulation of LC3-II was markedly increased in PLD + pUH + CQ group and in CQ group. These findings reflected the late-stage inhibition of autophagy by CQ. On the other hand, it was observed that the expression of LC3-II was slightly decreased in PLD + pUH group comparing to control group. Autophagy is usually induced by hyperthermic treatment as a response to protect cell from metabolic stress^26,27^. Nevertheless, there are controversy about how doxorubicin affects autophagy^28,29^. It is suggested that doxorubicin stimulates the initiation of autophagy but interferes the lysosomal function, therefore resulting in overall decrease in autophagy flux^29^. Park *et al*. demonstrated that doxorubicin reduces autophagosome formation via increase in mTOR expression^30^. The slightly reduced expression in LC3-II observed in PLD + pUH group could be explained by the summation of two opposite effects: upregulation by hyperthermia and downregulation by doxorubicin.

In our study, we used PLD + pUH to treat the tumors and employed CQ to prevent the remnant cancer cells escaping through autophagy. The results showed that when CQ used in combination with PLD + pUH, it assisted tumor suppression and postponed or even prevented tumor relapse. But CQ monotherapy has little impact on tumor growth as compared to the control group (as shown in Fig. 3B). Autophagy works as a surviving mechanism when cancer cells face strong stresses. CQ blocks autophagy in cancer cells damaged by anti-tumor therapy, and therefore aggravates the severe condition remaining cancer cells confront, and eventually results in decreasing the probability of tumor relapse. However, this effect is not prominent by CQ monotherapy, and hence in the absence of antitumor therapeutics CQ does not alter tumor growth response.

The survival rate in the PLD + pUH + CQ group was better than that in the PLD + pUH group. Observed up to Day 60, the survival rate is 75% (6 among 8 treated mice) and 56% (5 among 9 treated mice) for the PLD + pUH + CQ group and the PLD + pUH group, respectively. There was no observable tumor (complete remission) for these surviving mice after the treatment of PLD + pUH with or without CQ. Despite the great success in achieving long-term remission, the survival in the PLD + pUH + CQ group was not significantly better than PLD + pUH (p-value: 0.2). It might be due to that the therapeutic efficacy of PLD + pUH was sufficiently potent in suppressing tumor growth in the subcutaneous 4T1 tumor model, so the marginal benefit of adding CQ into treatment were harder to be demonstrated since PLD + pUH already had a good outcome. The difference of survival owing to CQ might be more apparent in a more lethal tumor model refreactory to PLD + pUH treatment. The survival in CQ group was nearly identical to that in control group, consistent with the observation in tumor growth that CQ monotherapy had little benefit.

CQ in cooperation with nanomedicine has been suggested as a promising therapeutic strategy to treat cancer^31^. Pelt *et al*. highlighted in their review the advantages of CQ to complement nanomedicine for cancer therapy, including autophagy inhibition, normalization of tumor vasculature, and reducing the hepatic clearance of nanoparticles^31^. There are increasing studies that practically exploit this strategy in cancer therapeutics. Sun *et al*. used CQ to reduce the ‘stemness’ of breast cancer stem cell to increase their susceptibility to chemotherapeutics such as doxorubicin and docetaxel^32^. Shao *et al*. designed a MPEG-PLA nanoparticle co-delivering CQ and doxorubicin to kill ovarian cancer, utilizing the lysosome-interfering property of CQ to hinder drug sequestration^33^. Wolfram *et al*. pretreated mice with CQ to reduce nanoparticle uptake by macrophage^34^. Lv *et al*. took advantage of the vessel-normalizing ability of CQ to improve microcirculation in tumor and promoted nanodrug delivery^35^. These studies demonstrated the versatile powerfulness of CQ in combination use with nanomedicine in cancer therapeutics. Our study further extended the strategy by adding pulsed-wave ultrasound hyperthermia, which assisted the delivery of nanodrug into tumor tissue and created a vicious microenvironment so that autophagy inhibition by CQ became crucial. To the best of our knowledge, this is the first paper to combine these three elements into an integrated strategy to successfully treat cancer and prolong remission.

## Conclusion

We demonstrated that pulsed-wave ultrasound hyperthermia (pUH) enhanced PEGylated liposomal doxorubicin (PLD) delivery in combination with chloroquine (CQ) could long-term suppress 4T1 tumor growth and postpone its recurrence. These results may pave the way to develop new combinatorial strategy for treatment-refractory cancer.

## Supplementary information

Supplementary Materials

## Data Availability

The data that support the findings of this study are available from the corresponding author upon reasonable request.

## References

[1] Kilonsky DJ (2007). Autophagy: from phenomenlogy to molecular understanding in less than a decade. Nat. Rev. Mol. Cell Biol..

[2] Amaravadi R, Kimmelman AC, White E (2006). Recent insights into the function of autophagy in cancer. Genes Dev..

[3] White E (2012). Deconvoluting the context-dependent role for autophagy in cancer. Nat. Rev. Cancer.

[4] Galluzzi L (2015). Autophagy in malignant transformation and cancer progression. EMBO J..

[5] Levy JM, Thorburn A (2011). Targeting autophagy during cancer therapy to improve clinical outcomes. Pharmacol. Ther..

[6] Levy JM, Towers CG, Thorburn A (2017). Targeting autophagy in cancer. Nat. Rev. Cancer.

[7] Gupta A (2010). Autophagy inhibition and anti-malarials promote cell death in gastrointestinal stromal tumor (GIST). Proc. Natl. Acad. Sc.i USA.

[8] Balic A (2014). Chloroquine targets pancreatic cancer stem cells via inhibition of CXCR4 and hedgehog signaling. Mol. Cancer Ther..

[9] Maes H (2014). Tumor vessel normalization by chloroquine independent of autophagy. Cancer Cell.

[10] Geser A, Brubaker G, Draper CC (1989). Effect of a malaria suppression program on the incidence of African Burkitt’s lymphoma. Am. J. Epidemiol..

[11] Hahn GM, Braun J, Har-Kedar I (1975). Thermochemotherapy: synergism between hyperthermia (42-43 degrees) and adriamycin (of bleomycin) in mammalian cell inactivation. Proc. Natl. Acad. Sci. USA.

[12] Thanou M, Gedroyc W (2013). MRI-guided focused ultrasound as a new method of drug delivery. J. Drug Deliv..

[13] Lizzi FL, Ostromogilsky M (1987). Analytical modelling of ultrasonically induced tissue heating. Ultrasound Med. Biol..

[14] Wu SK (2014). Short-time focused ultrasound hyperthermia enhances liposomal doxorubicin delivery and antitumor efficacy for brain metastasis of breast cancer. Int. J. Nanomedicine.

[15] Wu SK (2017). Pulsed-wave low-dose ultrasound hyperthermia selectively enhances nanodrug delivery and improves antitumor efficacy for brain metastasis of breast cancer. Ultrason. Sonochem..

[16] Tsang YW (2019). Modulated electro-hyperthermia-enhanced liposomal drug uptake by cancer cell. Int. J. Nanomedicine.

[17] Hirsch HA, Iliopoulos D, Tsichlis PN, Struhl K (2009). Metformin selectively targets cancer stem cells, and acts together with chemotherapy to block tumor growth and prolong remission. Cancer Res..

[18] Hirsch HA, Iliopoulos D, Struhl K (2013). Metformin inhibits the inflammatory response associated with cellular transformation and cancer stem cell growth. Proc. Natl. Acad. Sci. USA.

[19] King MA, Ganley IG, Flemington V (2016). Inhibition of cholesterol metabolism underlies synergy between mTOR pathway inhibition and chloroquine in bladder cancer cells. Oncogene.

[20] Choi DS (2014). Chloroquine eliminates cancer stem cells through deregulation of Jak2 and DNMT1. Stem Cells.

[21] Wei MF (2014). Autophagy promotes resistance to photodynamic therapy-induced apoptosis selectively in colorectal cancer stem-like cells. Autophagy.

[22] Lee SW (2015). The synergistic effect of combination temozolomide and chloroquine treatment is dependent on autophagy formation and p53 status in glioma cells. Cancer Lett..

[23] Maycotte P (2014). STAT3-mediated autophagy dependence identifies subtypes of breast cancer where autophagy inhibition can be efficacious. Cancer Res..

[24] Elliott IA (2019). Lysosome inhibition sensitizes pancreatic cancer to replication stress by aspartate depletion. Proc. Natl. Acad. Sci. USA.

[25] Schaaf MB (2019). Lysosomal pathways and autophagy distinctively control endothelial cell behavior to affect tumor vasculature. Front. Oncol..

[26] Yang W (2018). Combined treatment with modulated electro-hyperthermia and an autophagy inhibitor effectively inhibit ovarian and cervical cancer growth. Int. J. Hyperthermia.

[27] Ba MC (2017). Mild Hyperthermia enhances sensitivity of gastric cancer cells to chemotherapy through reactive oxygen species-induced autophagic death. Tumor Biol..

[28] Bartlett JJ, Trivedi PC, Pulinilkunnil T (2017). Autophagic dysregulation in doxorubicin cardiomyopathy. J. Mol. Cell Cardiol..

[29] Koleini N, Kardami E (2017). Autophagy and mitophagy in the context of doxorubicin-induced cardiotoxicity. Oncotarget.

[30] Park JH (2016). Doxorubicin Regulates Autophagy Signals via Accumulation of Cytosolic Ca(2+) in Human Cardiac Progenitor Cells. Int. J. Mol. Sci..

[31] Pelt J (2018). Chloroquine and nanoparticle drug delivery: A promising combination. Pharmacol. Ther..

[32] Sun R (2016). Nanoparticle-facilitated autophagy inhibition promotes the efficacy of chemotherapeutics against breast cancer stem cells. Biomaterials.

[33] Shao M (2018). Encapsulation of chloroquine and doxorubicin by MPEG-PLA to enhance anticancer effects by lysosomes inhibition in ovarian cancer. Int. J. Nanomedicine.

[34] Wolfram J (2017). A chloroquine-induced macrophage-preconditioning strategy for improved nanodelivery. Sci. Rep..

[35] Lv T (2018). Chloroquine in combination with aptamer-modified nanocomplexes for tumor vessel normalization and efficient erlotinib/Survivin shRNA co-delivery to overcome drug resistance in EGFR-mutated non-small cell lung cancer. Acta Biomater..

