# Supplementary material for: Pulsed-wave Ultrasound Hyperthermia Enhanced Nanodrug Delivery Combined with Chloroquine Exerts Effective Antitumor Response and Postpones Recurrence
Source: Sci Rep. 2019 Aug 28;9:12448. doi: 10.1038/s41598-019-47345-8 (PMC6713759; doi:10.1038/s41598-019-47345-8)
Supplement: Supplementary file 1 — Supplementary Materials [file 41598_2019_47345_MOESM1_ESM.pdf]

Supplementary Materials

Pulsed-wave Ultrasound Hyperthermia Enhanced Nanodrug Delivery Combined with Chloroquine Exerts Effective Antitumor Response and Postpones Recurrence

Chi-Feng Chiang[1], Yu-Hone Hsu[2], Chih-Chun Liu[1,3], Po-Chin Liang[1,4], Shi-Chuen Miaw[3], Win-Li Lin[1,5*]

[1]Department of Biomedical Engineering, National Taiwan University, Taipei, Taiwan

[2]Division of Neurosurgery, Department of Surgery, Kaohsiung Veterans General Hospital, Kaohsiung, Taiwan

[3]Graduate Institute of Immunology, College of Medicine, National Taiwan University, Taipei, Taiwan

[4]Department of Radiology, Department of Medical Imaging, National Taiwan University Hospital, Taipei, Taiwan

[5]Institute of Biomedical Engineering and Nanomedicine, National Health Research Institutes, Miaoli, Taiwan

Corresponding Author:

Win-Li Lin, PhD, Professor

Department of Biomedical Engineering, College of Medicine and College of Engineering, National Taiwan University, No.1, Sec.1, Jen-Ai Road, Taipei, Taiwan 100.

Phone: +886 2 23123456-81445; Fax: +886 2 23940049

Email: winli@ntu.edu.tw

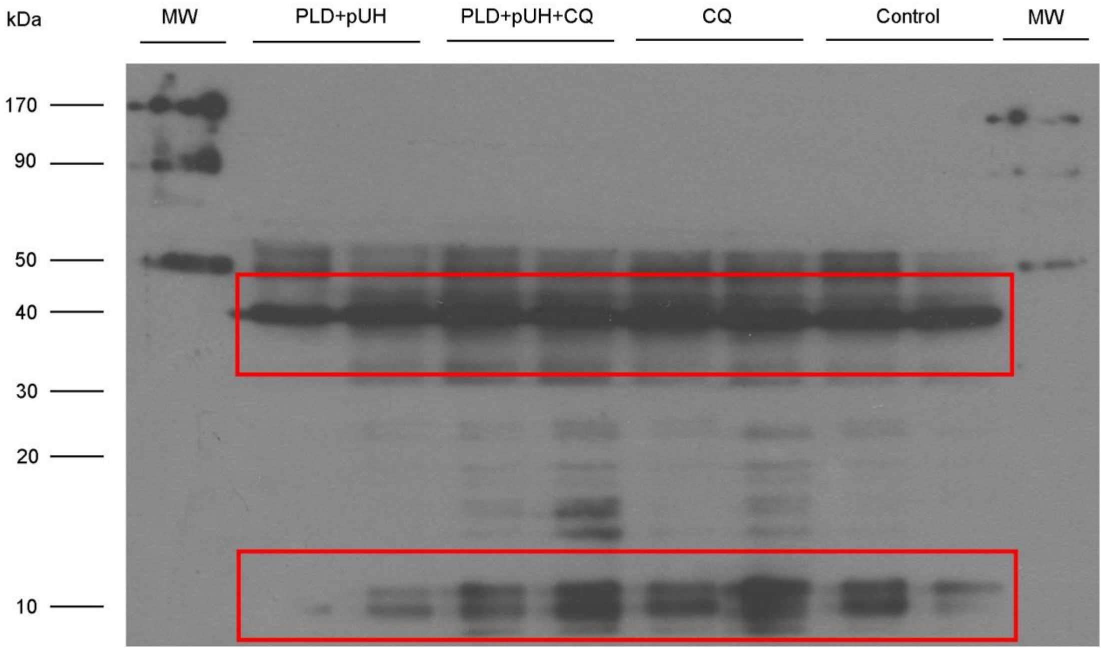

Supplementary Figure S1. Original full-length Western blot for the cropped image in Figure 7. The regions shown in Figure 7 were highlighted with red boxes. The upper box indicated the beta-actin bands, and the lower box indicated LC3-II bands.

Fluorescence staining for in vitro investigation of PLD uptaken by 4T1 breast tumor cells with or without CQ.

Purpose: To elucidate if administration of CQ changed the distribution of PLD, we designed an *in vitro* experiment to investigate the internalization of PLD enhanced by hyperthermia with/without CQ. Since the pharmacologic entity of PLD, doxorubicin, exercise its action by intercalating DNA, what really matters is the amount of doxorubicin brought into nucleus. Therefore we studied the fluorescence of doxorubicin within nucleus region, which could be distinctly depicted with Hoechst 33342 stain.

Materials and Methods: First, 4T1 murine breast cancer cells were seeded onto slide glasses (placed in 10-cm culture dish), supplied with RPMI 1640 medium containing 10% fetal bovine serum, and incubated for 24 hours. Then culture medium containing PLD solutions (0.01 mg/mL) with/without CQ (10μM) were added into culture dishes with seeded slides. Dishes with slides were immersed in 43℃ water bath for 5 minutes and then incubated for 48 hours. Slides were fixed with 4% formaldehyde solution and permeabilized with 20 μg/mL proteinase K and 0.2% Triton X-100 in PBS, and then stained with a solution containing Hoechst 33342 dye and mounted. Fluorescence images were obtained using a confocal microscope (AxioImager M1; Carl Zeiss Ltd., Oberkochen, Germany). Doxorubicin fluorescent detection was carried out with a green excitation/red emission filter, and blue channel for locating cell nuclei, respectively. All images were captured using the same exposure time. The pictures were merged using AxioVision Rel. 4.8 software (Carl Zeiss Ltd., Oberkochen, Germany). The fluorescent intensity of doxorubicin within nucleus regions depicted by Hoechst 33342 stain was measured by ImageJ software. The mean fluorescent intensity was derived by dividing measured fluorescence divided with nucleus regions area. For the sake of statistical analysis, five representative fields were selected from each slide for the calculation of mean fluorescent intensity.

Results and Discussion: Figure S2A showed the fluorescent microscopic images revealing the distribution of doxorubicin (red) in PLD+CQ+H/PLD+H groups with respect to cell nuclei (blue). Figure S2B represented the mean fluorescent intensity in PLD+CQ+H/PLD+H groups. The mean fluorescent intensity in PLD+CQ+H group was slightly higher than that in PLD+H group, but the difference is small and statistically insignificant ($p=0.743$). We concluded that CQ might enhance the transportation of PLD to nucleus, but this effect did not seem to be a key component underlying the extra anti-tumor efficacy of CQ in addition to PLD+pUH.

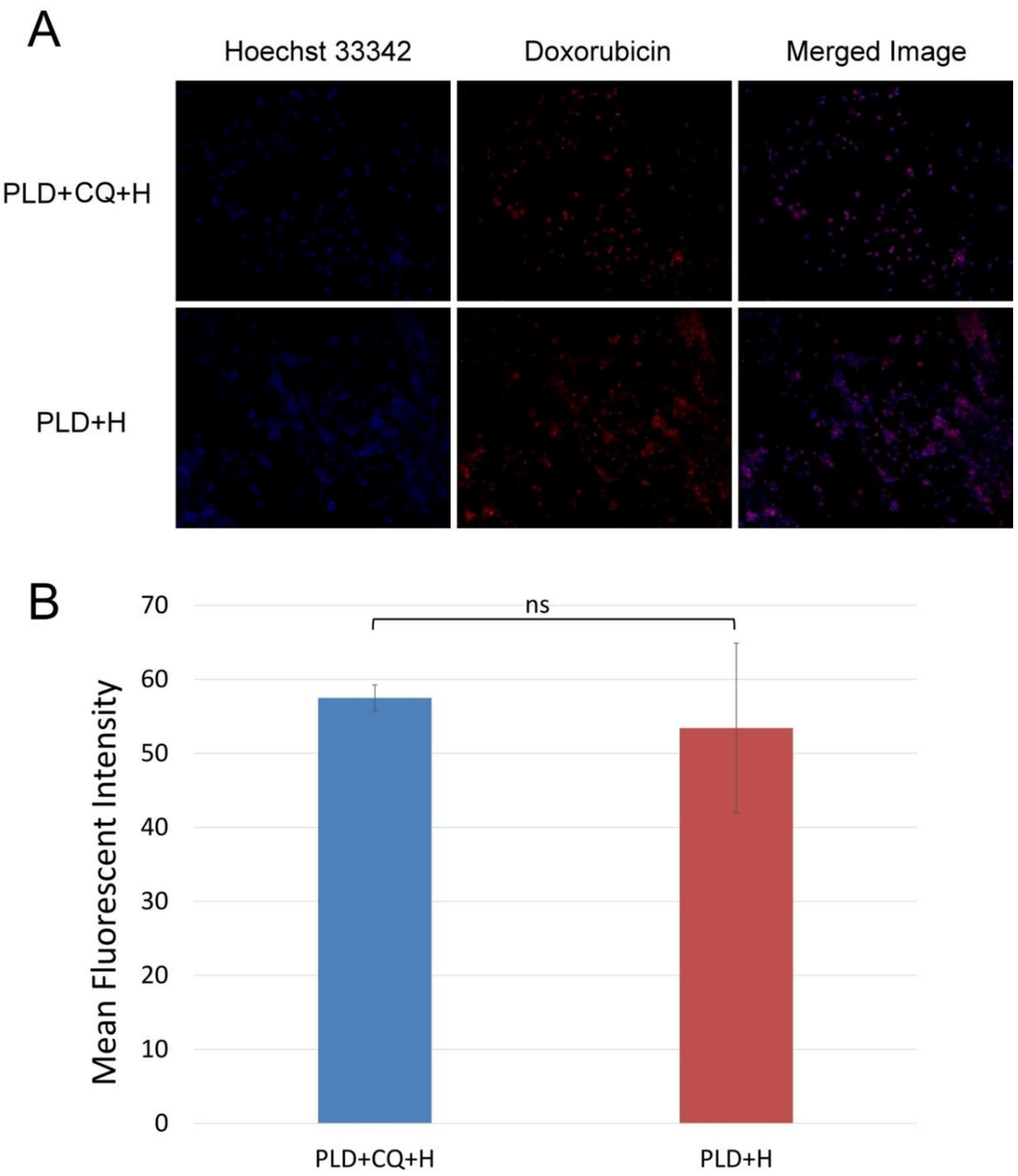

Supplementary Figure S2. (A) Fluorescent microscopic images of 4T1 murine breast cancer cells in vitro treated with PLD+CQ+H or PLD+H. Doxorubicin (red) distribution with respect to nuclei (blue, stained with Hoechst 33342 dye) were shown. (B) Mean fluorescent intensity of doxorubicin with respect to nucleus region area. ns: not significant.
